# Diagnostic value of symptoms and laboratory data for pertussis in adolescent and adult patients

**DOI:** 10.1186/1471-2334-13-129

**Published:** 2013-03-11

**Authors:** Naoyuki Miyashita, Hiroto Akaike, Hideto Teranishi, Yasuhiro Kawai, Kazunobu Ouchi, Tadashi Kato, Toshikiyo Hayashi, Niro Okimoto

**Affiliations:** 1 Department of Internal Medicine I, Kawasaki Medical School, 2-1-80 Nakasange, Kita-ku, Okayama, 700-8505, Japan; 2 Department of Pediatrics, Kawasaki Medical School, 2-1-80 Nakasange, Kita-ku, Okayama, 700-8505, Japan

**Keywords:** Bordetella pertussis, Adult, Adolescent, Seasonality, Fractional exhaled nitric oxide

## Abstract

**Background:**

Several symptoms are classically thought to be suggestive of pertussis in children, but the diagnostic value of these symptoms in adolescent and adult patients is unclear. We evaluated the accuracy of the clinical findings for the early presumptive diagnosis of pertussis in adolescent and adult patients. Furthermore, we measured fractional exhaled nitric oxide (FeNO) with regard to whether we could distinguish eosinophilic inflammation of the airway and pertussis. FeNO is not expected to be associated with pertussis.

**Methods:**

We compared 183 cases with laboratory-confirmed pertussis using serology and polymerase chain reaction and 1,132 cases without laboratory-confirmed pertussis.

**Results:**

Among pertussis patients, paroxysmal cough was common with 90% sensitivity, but the specificity was low (25%). Posttussive vomiting and whoop were less common (sensitivity 25% and 19%, respectively), but both showed greater specificity for pertussis (80% and 86%, respectively). Posttussive gagging was observed with intermediate frequency and provided greater specificity (49% and 77%, respectively). Pertussis cases were most frequent between May and August with a peak in June. The mean FeNO value for the pertussis patients was 18.2 ± 9.2 ppb, which was significantly lower than that in asthma patients (56.9 ± 20.3 ppb, *p* <0.001). The most useful definition was posttussive vomiting and/or gagging, and a plus normal FeNO value, which had a sensitivity of 72% and a specificity of 70%.

**Conclusions:**

Clinical symptoms and laboratory data are of limited value in making the diagnosis of pertussis, and it was clinically difficult to differentiate adolescent and adult patients with or without pertussis. However, pertussis should be considered if patients have posttussive vomiting and/or gagging and a normal FeNO concentration.

## Background

There has been a change in the age-related epidemiology of pertussis with an increasing proportion of cases reported among adolescent and adult patients in the United States, Canada, France and Australia, which all have longstanding vaccine-induced pertussis control [1]. A number of studies have documented that between 13% and 32% of adolescent and adult patients with an illness involving a cough of 6 days’ duration or longer have serologic evidence of pertussis [2-6]. However, pertussis in adults may be overlooked, because symptoms are often atypical, patients tend to present for medical attention after they have been coughing for some time, and nasopharyngeal cultures rarely are found to be positive. In addition, residual immunity from prior vaccination may modify the clinical presentation of pertussis in adolescent and adult patients, making the diagnosis even more difficult.

Rapid and simple diagnostic tests are useful tools in the early presumptive diagnosis of respiratory tract infections (RTI) caused by some pathogens. These results can be used to initiate appropriate antibiotic treatment and prevent outbreaks. The most widely used and quickest diagnostic method is the evaluation made by a physician based on clinical and laboratory findings. Several symptoms, such as paroxysmal cough, posttussive vomiting and inspiratory whoop are classically thought to be suggestive of pertussis in children [2], but the diagnostic value of each of these symptoms is uncertain in adolescent and adult patients. Relatively few studies have assessed the sensitivity and specificity of these classical symptoms [7]. The purpose of the present study was to identify a means of rapidly distinguishing pertussis from other causes of illness involving cough in daily clinical practice without waiting for serological or culture results. We evaluated the accuracy of the clinical findings for the early presumptive diagnosis of pertussis in adolescent and adult patients. Asthma and cough variant asthma are the leading causes of persistent and chronic cough and are thought to be the most important causes in the differential diagnosis of cough illness [8-10]. Because the measurement of fractional exhaled nitric oxide (FeNO) which reflects eosinophilic inflammation of the airway is simple, rapid, non-invasive and easy to perform and is characterized by high reproducibility, it may be considered as a good candidate for a screening test in the diagnosis of asthma and asthma-like syndromes [11,12]. To exclude the eosinophilic inflammation of the airway in daily clinical practice, we measured the FeNO concentration with regard to whether we could distinguish asthma and pertussis patients.

## Methods

### Study populations

Adolescent and adult patients with cough symptoms who visited Kawasaki Medical School Hospital, Kawasaki Medical School Kawasaki Hospital and Kurashiki Daiichi Hospital, from April 2005 to March 2012 were enrolled in this study. Underlying conditions that induce cough, especially chronic lung diseases, might influence the symptoms and laboratory data for pertussis in adolescent and adult patients. Thus, patients taking angiotensin-converting enzyme inhibitors or with known or suspected immunodeficiency, lung cancer, pneumonia, tuberculosis, non-tuberculous mycobacterial disease, influenza, chronic obstructive pulmonary diseases, bronchiectesis, diffuse panbronchiolitis, interstitial lung diseases, chronic sinusitis, gastroesophageal reflux, allergic rhinorhitis or asthma were excluded. Control subjects without complaints of cough were selected from healthy blood donors during the study period [13].

Determination of FeNO has been suggested to have a useful role in the evaluation of patients with chronic cough [14-16]. This study was conducted as part of cough studies investigating the usefulness of FeNO measurement for the differentiation of cough illness. FeNO was measured in pertussis patients and in age- and gender-matched healthy subjects who were selected from the medical staff and patients with asthma who regularly visited our hospital. A definitive diagnosis of bronchial asthma and cough variant asthma was made by a respiratory specialist in the outpatient clinic based on respiratory function, bronchial responsiveness, blood tests and treatment course in accordance with the Global Initiative for Asthma guidelines [17]. Informed consent was obtained from all patients, and the study protocol was approved by the Ethics Committee at Kawasaki Medical School.

We used a standardized questionnaire for collecting clinical information. Information on patient background, clinical signs and symptoms collected included paroxysmal cough, posttussive vomiting, posttussive gagging, inspiratory whoop, nocturnal cough, sputum production, chest pain, dyspnea, history of fever, upper RTI symptoms (sneeze, sore throat, nasal mucus, nasal congestion and headache) and history of cough in family members or work/school colleagues.

### Microbiological laboratory tests

Microbiological tests, such as Gram stain, cultures, real-time polymerase chain reaction (PCR) and serological tests, were performed as described previously [6,18]. Nasopharyngeal swab specimens were obtained from all patients and, if sputum was available, a Gram stain test, a quantitative culture and PCR were obtained. All nasopharyngeal swab specimens and sputum samples were also used for PCR and culturing. The target DNA sequences for PCR were a region of the insertion sequence IS*481* for *B. pertussis*[19], region of the 53-kDa gene for *C. pneumoniae*, the major outer membrane gene for *Chlamydia*, the P1 cytadhesin gene for *M. pneumoniae*, and the nucleotide sequence of the 5S-ribosomal DNA for *Legionella*. DNA was extracted from respiratory samples using a QIAamp DNA Mini Kit (QIAGEN K. K., Tokyo, Japan) in accordance with the manufacturer’s instructions. The assays were performed as described previously [6,18,20]. Cultures for *M. pneumoniae* and *Legionella* species were performed on pleuropneumonia-like organism broth (Difco, Detroit, MI, USA) and buffered charcoal-yeast extract alpha agar, respectively. Cultures for *Chlamydophila pneumoniae* and *C. psittaci* were performed using cycloheximide-treated HEp-2 cells grown in a 24-well cell culture plate [6]. All specimens were examined twice. Culture confirmation was done by fluorescent-antibody staining with *C. pneumoniae* and *C. psittaci* species-specific and genus-specific monoclonal antibodies [6]. When physicians suspected influenza, nasopharyngeal swab specimens were also tested for influenza A and B viruses by a direct enzyme immunoassay.

Paired serum samples were collected at intervals of at least 4 weeks after onset. Serum samples were tested for antibodies to *B. pertussis* pertussis toxin (PT) using an enzyme-linked immunosorbent assay method (Wako Chemicals, Tokyo, Japan) [21]. Antibodies against *M. pneumoniae* were measured with the use of a particle agglutination (PA) test (Serodia-Myco II kit, Fujirebio, Tokyo, Japan), *Legionella* species by a microagglutination test (detection of *L. pneumophila* serogroups 1 ~ 6, *L. bozemanii*, *L. dumoffii*, *L. gormanii*, and *L. micdadei*), *Coxiella burnetii* by an indirect immunofluorescence test, and viruses (influenza A and B viruses, adenovirus, respiratory syncytial virus, cytomegalovirus, and parainfluenza virus types 1, 2, and 3) by a complement fixation test. A microimmunofluorescence test was used for the titration of IgG and IgM antibodies against chlamydial species using formalinized elementary bodies of *C. pneumoniae* KKpn-15, *C. trachomatis* L2/434/Bu, and *C. psittaci* Budgerigar-1 strains as antigens [6]. Rheumatoid factors were absorbed with GullSORB (Meridian Bioscience Inc., OH, USA) before IgM titration.

### Criteria for the determination of microbial etiology

The microbial etiology was classified as “definitive”, “presumptive”, or “unknown” as reported previously [18]. A definitive etiology was recorded if one of the following conditions was present: (1) respiratory specimen culture or PCR results positive for *M. pneumoniae, C. psittaci* or *Legionella* species; (2) a fourfold increase in the antibody titer for viruses, *M. pneumoniae* (to ≥1:160), *Legionella* species (to ≥1:128), *C. burnetii*, or *Chlamydia* species (IgM or IgG); (3) a single increase in IgM titer for *Chlamydia* species ≥1:32; or (4) nasopharyngeal antigen test results positive for influenza A and B viruses. A presumptive etiology was considered if any of the following conditions were present: (1) an organism showing heavy (≥10^7^ cfu/mL) or moderate (10^6^ cfu/mL) growth of a predominant bacterium on a sputum culture in combination with Gram stain findings; (2) an antibody titer of ≥1:320 for *M. pneumoniae* in either an acute-phase or convalescent-phase serum samples; (3) an antibody titer of ≥1:256 for *Legionella* species in either an acute-phase or convalescent-phase serum samples; or (4) respiratory specimen culture or PCR results positive for *C. pneumoniae*.

Laboratory confirmation of recent infection with *B. pertussis* was defined if one of the following conditions was present: (1) a fourfold increase in anti-PT antibody; (2) a single serum anti-PT titer more than 3 standard deviations (SD) greater than the geometric mean of age-matched control values in either an acute-phase or convalescent-phase serum sample; or (3) nasopharyngeal swab PCR assay result positive. Using the criterion for the titer of a single PT antibody of ≥3 SD of the mean of 318 age-matched control values, the present results demonstrated that a level of ≥100 EU/mL defined a recent pertussis infection.

### Measurement of FeNO

The concentration of nitric oxide (NO) in expired air was evaluated on-line using a chemiluminescence analyzer NOA 280i (Sievers Instruments, Inc, USA). The measurement conditions of the on-line method were set in accordance with the American Thoracic Society and the European Respiratory Society guidelines [22]. From patients resting in a sitting position, expired gas after maximum inspiration was collected in a Mylar bag at an expiratory resistance of 5 cm H_2_O or higher and expiratory flow rate of 0.35 L/sec (0.315 - 0.385 L/sec). The measurement was performed in triplicate, and the mean was adopted.

### Statistical analysis

Statistical analysis was performed using Stat View version 5.0. (SAS Institute Inc, Cary, NC, USA). The incidence of clinical findings of patients with and without laboratory evidence of pertussis was compared using Fisher’s exact test. The mean age of patients, laboratory data and FeNO value were compared using Student’s *t* test.

## Results

### Patient characteristics

During the study period, 7,112 patients visited our hospital for the complaint of cough and 4,125 were excluded according to the exclusion criteria. Of the remaining 2,987 patients, physicians could not follow-up or could not carry out all microbiological tests in 1,662 patients. Finally, 1,325 patients were assessed using all the microbiological tests described in the Methods section. Cough duration of these patients at first visit was 3 to 21 days after onset of disease (mean 14.1 days). Among these cough patients, we excluded from the analysis 10 cases of mixed-pertussis infection with other pathogens. Finally, we enrolled and analyzed 183 cases of laboratory-confirmed pertussis. Of these, 36 cases demonstrated a fourfold increase in antibody titer (16 cases were PCR positive), and 125 cases demonstrated a single serum titer ≥3 SD above the mean of the control values, and 41 cases were PCR positive (19 cases were positive for both tests).

Table 1 shows the characteristics of the patients with and without laboratory-confirmed pertussis. No significant differences in age or gender were identified between the two groups, but patients with laboratory-confirmed pertussis were more likely to have paroxysmal cough, posttussive gagging and inspiratory whoop. The proportion of patients with a family member or work/school colleague with a coughing illness differed between the laboratory-confirmed pertussis and no laboratory-confirmed pertussis groups (53% vs 24%, *p* <0.0001). No significant differences in laboratory data were found between the two groups, and mean WBC and mean lymphocyte counts were normal in both groups.

**Table 1:** Characteristics of patients with and without laboratory-confirmed pertussis

**Characteristics**	**Laboratory-confirmed pertussis**	**No laboratory-confirmed pertussis**	***p*- value**
Number	183	1132	
Age mean (range), years	39.1 (16–77)	37.6 (16–79)	0.3189
Male : Female	73 : 110	501 : 631	0.2965
Symptom*			
Paroxysmal cough	165 (90.1)	849 (75.0)	<0.0001
Awakened by cough	133 (72.6)	747 (65.9)	0.0760
Sputum production	67 (36.6)	378 (33.3)	0.4006
Chest pain	66 (36.0)	382 (33.7)	0.5566
Dyspnea	56 (30.6)	297 (26.2)	0.2423
Posttussive vomiting	46 (25.1)	228 (20.1)	0.1407
Posttussive gagging	90 (49.1)	259 (22.8)	<0.0001
Inspiratory whoop	36 (19.6)	156 (13.7)	0.0420
History of fever (≥37.0°C)	27 (14.7)	234 (20.6)	0.0718
Upper respiratory tract infection symptoms before cough	83 (45.3)	561 (49.5)	0.3011
Exposed to cough by family member or work/school colleague*	97 (53.0)	273 (24.1)	<0.0001
Hospitalization*	1 (0.5)	10 (0.8)	>0.9999
Laboratory data			
WBC mean number ± SD, /mm^3^	6,619 ± 2,815	7,034 ± 2,622	0.1925
Lymphocyte mean number ± SD, /mm^3^	2,043 ± 892	2,211 ± 973	0.1244

*Data represent the numbers (%) of patients.

### Differentiation among laboratory-confirmed pathogens

Among the patients without laboratory-confirmed pertussis, other pathogens were established in 252 cases. *M. pneumoniae* was detected in 105 cases, *C. pneumoniae* in 93 cases, viruses in 52 cases and bacteria in 12 cases. Among viruses, influenza was excluded in this study because the clinical presentation of influenza is clearly different from respiratory infections with other pathogens. No cases with *C. psittaci*, *C. burnetii*, or *Legionella* species were detected. Among the *M. pneumoniae* cases, three were culture positive, 86 were PCR positive, and 43 demonstrated positive serological results (27 cases were positive for two methods). Among the *C. pneumoniae* cases, 59 were PCR positive and 42 demonstrated positive serological results (eight cases were positive for both methods).

Table 2 shows the characteristics of the patients with laboratory-confirmed pertussis, *M. pneumoniae*, *C. pneumoniae*, viruses and mixed-pertussis infection with other pathogens: *C. pneumoniae* (one case), *M. pneumoniae* (one case), *S. pneumoniae* (one cases), *Moraxella catarrhalis* (one case), and viruses (six cases). Characteristics were almost identical among patients with pertussis, *M. pneumoniae*, *C. pneumoniae*, and mixed-pertussis infection with other pathogens but patients with laboratory-confirmed pertussis were more likely to have posttussive gagging and inspiratory whoop. In contrast, many significant differences were observed between the laboratory-confirmed pertussis and viruses patients.

**Table 2:** **Characteristics of patients with laboratory-confirmed pertussis, *M. pneumoniae*, *C. pneumoniae*, viruses and mixed-pertussis infection with other pathogens**

**Characteristics**	**Pertussis**	***M. pneumoniae***	***C. pneumoniae***	**Viruses**	Mixed-pertussis infection*
Number	183	105	93	52	10
Age mean (range), years	39.1 (16–77)	35.2 (16–46)	36.4 (18–52)	39.4 (24–76)	39.7 (27–65)
Male : Female	73 : 110	49 : 56	45 : 48	25 : 27	5 : 5
Symptom**					
Paroxysmal cough	165 (90.1)	89 (84.7)	68 (73.1)***	24 (46.1)***	100 (100.0)
Awakened by cough	133 (72.6)	84 (80.0)	66 (70.9)	29 (55.7)***	9 (90.0)
Sputum production	67 (36.6)	40 (38.0)	30 (32.2)	17 (32.6)	5 (50.0)
Chest pain	66 (36.0)	38 (36.1)	25 (26.8)	12 (23.0)	3 (30.0)
Dyspnea	56 (30.6)	30 (28.5)	21 (22.5)	6 (11.5)***	4 (40.0)
Posttussive vomiting	46 (25.1)	20 (19.0)	15 (16.1)	0 ***	2 (20.0)
Posttussive gagging	90 (49.1)	32 (30.4)***	22 (23.6)***	6 (11.5)***	6 (60.0)
Inspiratory whoop	36 (19.6)	11 (10.4)***	10 (10.7)***	0***	1 (10.0)
History of fever (≥37.0°C)	27 (14.7)	35 (33.3)***	8 (8.6)	19 (36.5)***	50 (50.0)***
Upper respiratory tract infection symptoms before cough	83 (45.3)	55 (52.3)	37 (39.7)	45 (86.5)***	8 (80.0)***
Exposed to cough by family member or work/school colleague**	97 (53.0)	49 (46.6)	40 (43.0)	26 (50.0)	60 (60.0)
Hospitalization**	1 (0.5)	1 (0.9)	0	0	1 (10.0)
Laboratory data					
WBC mean number ± SD, /mm^3^	6,619 ± 2,815	6,249 ± 2,731	6,972 ± 2,618	6,314 ± 2,765	6,898 ± 2,265
Lymphocyte mean number ± SD, /mm^3^	2,043 ± 892	1,986 ± 878	2,361 ± 958	2,419 ± 954	2,133 ± 854

* Mixed-pertussis infection with other pathogens: *Chlamydophila pneumoniae* (one case), *Mycoplasma pneumoniae* (one case), *Streptococcus pneumoniae* (one cases), *Moraxella catarrhalis* (one case) and viruses (six cases).

** Data represent the numbers (%) of patients.

*** When compared to patients with laboratory-confirmed pertussis, the differences were significant (*p*-value indicate <0.05).

### Seasonality

**Figure 1:**
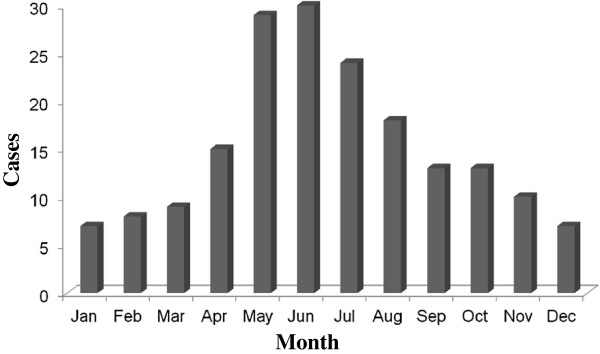
Cases of pertussis in adolescent and adult patients by month between 2005 and 2012.

During the period between 2005 and 2012, the average monthly number of sera and nasopharyngeal swabs submitted for serology and PCR was highest during the winter months of December through February. In contrast, positive pertussis cases were most frequent during the months May through August with a peak in June (Figure 1). Although annual fluctuations in the number of samples tested and the number of positive pertussis cases was observed during the study period, the peaks of pertussis cases were distributed between May and July in each year.

### FeNO concentration

We started the measurement of FeNO concentrations from 2007 routinely. Thus, we measured FeNO concentrations in 112 pertussis patients and compared these with 112 healthy volunteers and 112 asthma patients. The mean FeNO value for the pertussis patients was 18.2 ± 9.2 ppb. This value was significantly lower than that in asthma patients (56.9 ± 20.3 ppb, *p* <0.001) but not significantly different from that in healthy volunteers (16.4 ± 6.8 ppb, *p* = 0.2181).

## Discussion

There is increasing appreciation of pertussis as an unrecognized but important cause of prolonged cough illness in older age groups. In terms of the clinical symptoms of pertussis in adolescents and adults, De Serres et al. published data on the symptoms of 664 cases [23]. Among classic pertussis symptoms, the most common symptoms were paroxysmal cough (99%), next to posttussive apnea (87%), whoop (69%) and posttussive vomiting (65%). Von Koenig et al. reviewed symptoms described in studies in adult patients [3]. Although, the incidence of paroxysmal cough was similar among studies and ranged from 70% to 99%, whoop and posttussive vomiting exhibited wide variations with ranges from 8% to 82% and 17% to 65%, respectively. Subsequently, Rothstein and Edwards reviewed symptoms described in studies into health burden in adults [4]. Studies suggest that between 21% and 86% of adults have classic pertussis symptoms such as paroxysmal cough, whoop and posttussive vomiting.

Today, many different case definitions are used throughout the world. Ghanaie et al. studied the sensitivity and specificity of the WHO pertussis clinical case definitions in 328 children aged 6 to 14 years with persistent cough for ≥2 weeks [24]. The sensitivity was 95.2% and the specificity was 15.0% with cough ≥2 weeks plus one of the WHO clinical criteria. With an increasing number of clinical findings, sensitivity decreased and specificity increased. Posttussive vomiting was the symptom that had the most pronounced effect in increasing specificity. In a study of pertussis in adolescents and adults by Strebel et al. [5], the sensitivity and specificity of paroxysmal cough were 100% and 12%, respectively. With posttussive vomiting, the sensitivity was 56% and the specificity was 68%; for whooping, the sensitivity was 28% and the specificity was 85%. In 1988, Patriarca et al. evaluated 15 clinical case definitions for pertussis during community outbreaks and concluded that a definition of ≥2 weeks of cough was both sensitive (77–91%) and specific (54–71%) for monitoring culture-positive cases [25]. However, in nonoutbreak situations, the use of their case definitions had low sensitivity [26].

Among our adolescent and adult patients with pertussis, paroxysmal cough was common with 90% sensitivity, but the specificity of this finding was low (25%). Posttussive vomiting and whoop were less common (sensitivity 25% and 19%, respectively), but both showed greater specificity for pertussis infection (80% and 86%, respectively). Strebel et al. indicated that pertussis should be considered in the differential diagnosis of acute cough illness in adolescent and adult patients, especially if the cough is associated with posttussive vomiting and/or gagging [5]. Our results also demonstrated that patients with laboratory-confirmed pertussis were more likely to have a history of posttussive gagging, but the sensitivity was intermediate (sensitivity: 49%; specificity: 77%). Exposure history of coughing is one tool to differentiate patients with pertussis from those without the disease but sensitivity was intermediate (sensitivity: 53%; specificity: 76%). Our results together with the results of previous studies suggest that individual symptoms may be of limited help in distinguishing pertussis from other causes of cough [5,27].

To improve pertussis diagnosis, the Global Pertussis Initiative suggested a different approach [28]. They developed an algorithm that delineates the signs/symptoms of pertussis most common to three age groups: 0–3 months, 4 months to 9 years, and ≥10 years. In patients ≥10 years of age, the presence of a worsening paroxysmal, nonproductive cough of ≥7 days’ duration while afebrile but with coryza that has not become purulent would also indicate high sensitivity and good specificity for pertussis. In addition, the presence of sweating episodes between paroxysms significantly increase specificity. Determination of their utility and their sensitivity and specificity versus existing case definitions is required.

Seasonal increases are a common phenomenon in infectious diseases, but the underlying mechanisms are not entirely clear [29]. For respiratory pathogens, seasonal increases are thought to be driven by seasonal variations in survival of the pathogen outside the host, host behavior and the level of host immunity. Several studies have reported that pertussis has seasonality, with activity generally peaking in August [30,31] or between the early summer and early autumn [5,32,33]. Our study during the past 7 years demonstrated a clear seasonal pattern in the incidence of reported cases, with many cases occurring between May and August each year. Pertussis is specified for weekly reporting by specially designated sentinel pediatric clinics in accordance with the Japanese Infectious Diseases Control Law [34]. The same seasonal observations with peaks between May and July were reported in pediatric patients during the past decade [35]. In contrast to Western countries, April is the start of the school year for schools and universities in Japan. In several recent years, many outbreaks in schools and universities occurred between May and July [20,35]. Thus, the starting of schools and universities may play a role in seasonal increases in the incidence of pertussis in Japan.

To improve the diagnostic accuracy, we assessed the use of combinations of symptoms and other findings. The most useful combination definition was posttussive vomiting and/or gagging, and a normal FeNO value, which had a sensitivity of 72% and a specificity of 70%. The combination of symptoms and laboratory findings may be slightly more helpful.

Our study had several limitations. One of the serious concerns about our study is the exclusion of large number of patients with underlying diseases that caused persistent cough. Unfortunately, we did not perform microbiological tests in these excluded patients. However, pertussis could be present in these groups and data on the performance of the tests in these patients would be useful. Thus, further evaluation, including larger sample sizes with underlying diseases and cough patients, is necessary. Another limitation was that we did not carry out culture because several studies demonstrated the sensitivity of culture in adolescent and adult patients was extremely low. Thus, we used PCR instead of culture for the diagnosis of *B. pertussis*. In addition, the sample size for patients with laboratory-confirmed viruses was too small. In this study, we used only serological tests to identify viruses, thus, further studies including larger sample sizes of cough patients due to viruses identified using culture and PCR are necessary.

## Conclusions

We found a clear seasonal pattern in the occurrence of pertussis cases in adolescent and adult patients. Clinical symptoms and laboratory data were of limited value in making the diagnosis of pertussis and it was clinically difficult to differentiate patients with pertussis from those without the disease in adolescent and adult patients. However, cough is a common symptom and is treated symptomatically without a definitive diagnosis. Thus, pertussis should be considered in the differential diagnosis of cough illness in adolescent and adult patients, especially if the patient has posttussive vomiting and/or gagging and normal FeNO concentration.

## Competing interests

The authors declare that they have no competing interests.

## Authors’ contributions

NM, KO and NO conceived the study and participated in its design and coordination. NM, HA, HT, YK, TK, and TH collected and managed the data, including quality control, and carried out the microbiological laboratory tests. NM, KO and NO drafted the manuscript, and all authors contributed substantially to its revision. All the authors read and approved the final manuscript.

## Pre-publication history

The pre-publication history for this paper can be accessed here:

http://www.biomedcentral.com/1471-2334/13/129/prepub

## References

[B1] Halperin SA The control of pertusis-2007 and beyond N Engl J Med 2007 356 2 110 113 10.1056/NEJMp068288 17215528

[B2] Hewlett EL Edwards KM Pertussis-not just for kids N Engl J Med 2005 352 12 1215 1222 10.1056/NEJMcp041025 15788498

[B3] Von Koenig CHW Halperin S Riffelmann M Guiso N Pertussis of adults and infants Lancet Infect Dis 2002 2 12 744 750 10.1016/S1473-3099(02)00452-8 12467690

[B4] Rothstein E Edwards K Health burden of pertussis in adolescents and adults Pediatr Infect Dis J 2005 24 5 S44 S47 15876923 10.1097/01.inf.0000160912.58660.87

[B5] Strebel P Nordin J Edwards K Hunt J Besser J Burns S Amundson G Baughman A Wattigney W Population-based incidence of pertussis among adolescents and adults, Minnesota, 1995–1996 J Infect Dis 2001 183 9 1353 1359 10.1086/319853 11294666

[B6] Miyashita N Fukano H Yoshida K Niki Y Matsushima T *Chlamydia pneumoniae* infection in adult patients with persistent cough J Med Microbiol 2003 52 3 265 269 10.1099/jmm.0.04986-0 12621093

[B7] Cornia PB Hersh AL Lipsky BA Newman TB Gonzales R Does this coughing adolescent or adult patient have pertussis? JAMA 2010 304 8 890 896 10.1001/jama.2010.1181 20736473

[B8] Fujimura M Abo M Ogawa H Nishi K Kibe Y Hirose T Nakatsumi Y Iwasa K Importance of atopic cough, cough variant asthma and sinobronchial syndrome as causes of chronic cough in the Hokuriku area of Japan Respirology 2005 10 2 201 207 10.1111/j.1440-1843.2005.00686.x 15823186

[B9] Matsumoto H Niimi A Takemura M Ueda T Yamaguchi M Matsuoka H Jinnai M Chin K Mishima M Prevalence and clinical manifestations of gastro-oesophageal reflux-associated chronic cough in the Japanese population Cough 2007 3 1 1 10.1186/1745-9974-3-1 17210085 PMC1781074

[B10] Yamasaki A Hanaki K Tomita K Watanabe M Hasagawa Y Okazaki R Yamamura M Fukutani K Sugimoto Y Kato K Kodani M Ikeda T Konishi T Kawasaki Y Tokuyasu H Yajima H Sejima H Isobe T Shimizu E Cough and asthma diagnosis: physicians’ diagnosis and treatment of patients complaining of acute, subacute and chronic cough in rural areas of Japan Int J Gen Med 2010 3 4 101 107 20463827 10.2147/ijgm.s8167 PMC2866550

[B11] Barnes PJ Belvisi MG Nitric oxide and lung disease Thorax 1993 48 10 1034 1043 10.1136/thx.48.10.1034 7903007 PMC464825

[B12] Kharitonov SA Barnes PJ Exhaled markers of pulmonary disease Am J Respi Crit Care Med 2001 163 7 1693 1722 10.1164/ajrccm.163.7.2009041 11401895

[B13] Miyashita N Kawai Y Yamaguchi T Ouchi K Evaluation of serological tests for diagnosis of Bordetella pertussis infection in adolescents and adults Respirology 2011 16 8 1189 1195 10.1111/j.1440-1843.2011.02024.x 21790881

[B14] Chatokin JM Ansarin K Silkoff PE McClean P Gutierrez C Zamel N Chapman KR Exhaled nitric oxide as a noninvasive assessment of chronic cough Am J Respir Crit Care Med 1999 159 6 1810 1813 10351923 10.1164/ajrccm.159.6.9809047

[B15] Chaudhuri R McMahon AD Thomson LJ MacLeod KJ McSharry CP Livingston E McKay A Thomson NC Effect of inhaled corticosteroids on symptom severity and sputum mediator levels in chronic persistent cough J Allergy Clin Immunol 2004 113 6 1063 1070 10.1016/j.jaci.2004.03.019 15208586

[B16] Kowal K Bodzenta-Lukaszyk A Zukowski S Exhaled nitric oxide in evaluation of young adults with chronic cough J Asthma 2009 46 7 692 698 10.1080/02770900903056187 19728207

[B17] National Institute of Health, National Heart, Lung, and Blood Institute Global Initiative For Asthma. Global Strategy For Asthma Management And Prevention 2012 (update) 2008 1 128 http://www.ginasthm.org/documents/4

[B18] Miyashita N Kawai Y Yamaguchi T Ouchi K Oka M Atypical Pathogen Study Group Clinical potential of diagnostic methods for the rapid diagnosis of *Mycoplasma pneumoniae* pneumonia in adults Eur J Clin Microbiol Infect Dis 2011 30 3 439 446 10.1007/s10096-010-1107-8 21061035

[B19] Riffelmann M von Konig CH W Caro V Guiso N for the Pertussis PCR Consensus Group Nucleic acid amplification tests for diagnosis of *Bordetella* infections J Clin Microbiol 2005 43 10 4925 4929 10.1128/JCM.43.10.4925-4929.2005 16207944 PMC1248485

[B20] Miyashita N Kawai Y Yamaguchi T Ouchi K Kurose K Oka M Outbreak of pertussis in a university laboratory Intern Med 2011 50 8 879 885 10.2169/internalmedicine.50.4768 21498936

[B21] Kuno-Sakai H Kimura M Ohta K Oh Y Kim R Kobayashi T Yamamoto E Fujita I A simple and sensitive ELISA of antibodies to pertussis antigens Vaccine 1992 10 5 350 352 10.1016/0264-410X(92)90377-V 1574921

[B22] American Thoracic Society Documents ATS/ERS recommendations for standardized procedures for the online and offline measurement of exhaled lower respiratory nitric oxide and nasal nitric oxide, 2005 Am J Respir Crit Care Med 2005 171 8 912 930 15817806 10.1164/rccm.200406-710ST

[B23] De Serres G Shadmani R Duval B Boulianne N Dery P Fradet MD Rochette L Halperin SA Morbidity of pertussis in adolescents and adults J Infect Dis 2000 182 7 174 179 10882595 10.1086/315648

[B24] Ghanaie RM Karimi A Sadeghi H Esteghamti A Falah F Armin S Fahimzad A Shamshiri A Kahbazi M Shiva F Sensitivity and specificity of World Health Organization pertussis clinical case definition Int J Infect Dis 2010 14 12 e1072 e1075 10.1016/j.ijid.2010.07.005 20951620

[B25] Patriarca PA Biellik RJ Sanden G Burstyn DG Mitchell PD Silverman PR Davis JP Manclark CR Sensitivity and specificity of clinical case definitions for pertussis Am J Public Health 1988 78 7 833 836 10.2105/AJPH.78.7.833 3289409 PMC1350347

[B26] Stehr K Cherry JD Heininger U A comparative efficacy trial in Germany in infants who received either the Lederle/Takeda acellular pertussis component DTP (DTaP) vaccine, the Lederle whole-cell component DTP vaccine, or DT vaccine Pediatrics 1998 101 1 Pt 1 1 11 9417143 10.1542/peds.101.1.1

[B27] Park WB Park SW Kim HB Kim EC Oh M Choe KW Pertussis in adults with persistent cough in South Korea Eur J Clin Microbiol Infect Dis 2005 24 2 156 158 10.1007/s10096-005-1277-y 15711786

[B28] Cherry JD Tan T von Koenig CHW Forsyth KD Thisyakorn U Greenberg D Johnson D Marchant C Plotkin S Clinical definitions of pertussis: Summary of a global pertussis initiative roundtable meeting, February 2011 Clin Infect Dis 2012 54 12 1756 1764 10.1093/cid/cis302 22431797 PMC3357482

[B29] Altizer S Dobson A Hosseini P Hudson P Pascual M Rohani P Seasonality and the dynamics of infectious diseases Ecol Lett 2006 9 4 467 484 10.1111/j.1461-0248.2005.00879.x 16623732

[B30] De Greeff SC Dekkers AL Teunis P Rahamat-Langendoen JC Mooi FR De Melker HE Seasonal patterns in time series of pertussis Epidemiol Infect 2009 137 10 1388 1395 10.1017/S0950268809002489 19327200

[B31] Shah AP Smolensky MH Burau KD Cech IM Lai D Seasonality of primary childhood and young adult infectious diseases in the United States Chromob Int 2006 23 5 1065 1082 10.1080/07420520600920718 17050218

[B32] Skowronski DM De Serres G MacDonald D Wu W Shaw C Macnabb J Champagne S Patrick DM Harperin SA The changing age and seasonal profile of pertussis in Canada J Infect Dis 2002 185 10 1448 1453 10.1086/340280 11992280

[B33] Vickers D Maina-Jaime RC Pahwa P Pertussis in rural populations of Saskatchewan (1995 to 2003) Can J Pub Health 2006 97 6 459 464 17203725 10.1007/BF03405228 PMC6975707

[B34] National Institute of Health. Infectious Disease Surveillance Center Pertussishttp://www.nih.go.jp/niid/ja/10/2096-weeklygraph/1652-09pertus.html

[B35] National Institute of Health. Infectious Disease Surveillance Center Pertussis 2005–2007 IASR 2008 29 65 77 http://idsc.nih.go.jp/iasr/29/337/inx337-j.html

